# Non-immune Cell Components in the Gastrointestinal Tumor Microenvironment Influencing Tumor Immunotherapy

**DOI:** 10.3389/fcell.2021.729941

**Published:** 2021-10-13

**Authors:** Zhengshuo Li, Xiaoyue Zhang, Can Liu, Jian Ma

**Affiliations:** ^1^Hunan Cancer Hospital and the Affiliated Cancer Hospital of Xiangya School of Medicine, Central South University, Changsha, China; ^2^Cancer Research Institute and School of Basic Medical Science, Central South University, Changsha, China; ^3^Key Laboratory of Carcinogenesis and Cancer Invasion of the Chinese Ministry of Education, Changsha, China; ^4^NHC Key Laboratory of Carcinogenesis and Hunan Key Laboratory of Nonresolving Inflammation and Cancer, Hunan Key Laboratory of Cancer Metabolism, Hunan Key Laboratory of Translational Radiation Oncology, Changsha, China

**Keywords:** gastrointestinal cancer, tumor microenvironment, exosome, cancer associated fibroblasts, nerves, cytokines, microbiome, tumor immunotherapy

## Abstract

Interactions of genetic susceptibility factors, immune microenvironment, and microbial factors contribute to gastrointestinal tumorigenesis. The suppressive immune microenvironment reshaped by the tumors during gastrointestinal tumorigenesis directly contributes to T-cell depletion in tumor immunotherapy. Soluble factors secreted by tumor cells or stromal cells collectively shape the suppressive immune environment. Here, we reviewed the key factors in the gastrointestinal tumor microenvironment that influence tumor immunotherapy, focusing on the effects of fibroblasts, neuronal cells, soluble cytokines, exosomes, and the microbiome in tumor microenvironment. Research in this field has helped to identify more precise and effective biomarkers and therapeutic targets in the era of tumor immunotherapy.

## Introduction

Growing evidence suggests that the tumor microenvironment (TME) plays a crucial role in promoting or inhibiting tumor progression. TME consists of extracellular matrix, stromal cells (such as fibroblasts, mesenchymal stromal cells, endothelial cells, neurons, blood, and lymphatic network, etc.) and immune cells (including T and B lymphocytes, natural killer cells, and tumor-associated macrophages, etc.) (Hanahan and Coussens, 2012). As the tumor progresses, the metabolism of tumor cells is altered, producing hypoxia, oxidative stress, and a low pH value TME which filled with immune cells and soluble cytokines (Catalano et al., 2013). TME not only plays a key role in tumor initiation, progression and metastasis, but also has a profound impact on the effectiveness of immunotherapy. Tumor immunotherapy has achieved unprecedented success, however, some problems remain, such as low response rates, high immunotoxicity and drug resistance. Infiltration and activation status of immune cells in TME, such as CD8^+^ cell infiltration, macrophage polarization, B cell and dendritic cell antigen presentation efficiency, the degree of natural killer cell activation and chemotaxis of immunosuppressive cells [such as regulatory T (Treg) and myeloid-derived suppressor cells (MDSC)], could determine the efficiency of immunotherapy (Quante et al., 2013).

The chronic inflammation-induced tumorigenesis is partly mediated by immune cells and the cytokines they produce, which alter the microenvironment to support tumor formation and progression (Karin et al., 2006). Gastrointestinal cancers have more chronic inflammation due to constant interactions with microorganisms, resulting in a very active TME (Quante and Wang, 2008). In an inflammation-induced gastric cancer model, MDSCs cells are heavily infiltrated in the TME, and MDSCs resist anti-tumor immunity by disrupting T-cell function and promoting the differentiation of immunosuppressive Treg cells (Tu et al., 2008). Immunoscore of infiltrating T and B cells in colorectal cancer tumors can determine patient prognosis, and the predictive accuracy was superior to PD-L1 expression (Van den Eynde et al., 2018). Furthermore, fibroblasts and neuronal cells in the gastrointestinal TME can interact with immune cells in TME, and are able to influence almost all tumor malignant phenotypes (Saloman et al., 2016; Pereira et al., 2019). Colorectal cancer patients with high microsatellite instability (MSI) or mismatch repair-deficient (dMMR) are susceptible to checkpoint inhibitors (Oliveira et al., 2019). dMMR is associated in tumors with a high number of activated CD8^+^ cytotoxic t cells and upregulated immune checkpoints, providing a theoretical guide for PD-1 and PD-L1 blockade therapies in gastrointestinal tumors (Le et al., 2015).

**FIGURE 1:**
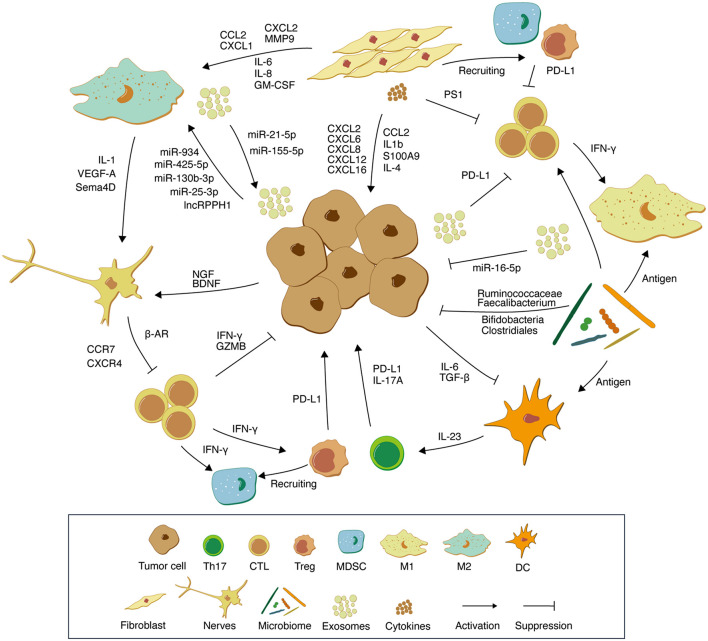
Schematic representation of the interactions between tumor cells and various components of the TME. Tumor-derived exosomes carrying PD-L1 molecules and multiple microRNAs inhibit M1-type macrophage and T cell proliferation. M2-type macrophages and tumor cells promote mutual growth through exosomal microRNAs and jointly mediate tumor immune escape. CAFs recruit suppressor immune cells such as MDSCs and Tregs by secreting multiple chemokines. In addition, CAFs promote tumor cell proliferation by secreting cytokines such as IL-1β, IL-4, and S100A9. NGF and BDNF in TME are able to increase neuronal cell abundance, which in turn leads to an increase in adrenergic signaling, resulting in the accumulation of norepinephrine and promoting tumor growth. The microbiome enhances anti-tumor immunity through antigen activation of DCs and M1-type macrophages. In addition, microflora beneficial for tumor immunotherapy such as *Bifidobacteria*, *Clostridiales*, *Ruminococcaceae*, and *Faecalibacterium* activate antitumor immunity through their metabolites. In TME, non-immune cell components create a “soil” suitable for tumor cell growth by communicating with immune cells and tumor cells. Th17, T helper cell 17; CTL, Cytotoxic T lymphocytes; Treg, Regulatory T cells; MDSC, Myeloid-derived suppressor cells; M1, M1-polarized macrophages; M2, M1-polarized macrophages; DC, Dendritic cells.

In this review, we focus on the non-immune cell components of the gastrointestinal (mainly stomach and colorectum) TME such as fibroblasts, neurons, cytokines, exosomes, metabolites, and microbiome, and discuss the main functions and potential roles played by these components in tumor immunity (Figure 1). Although these components are not directly involved in tumor growth, they play an important role in tumor immunotherapy by remodeling the TME to provide a stable ground for tumor cell growth. Combining existing studies and interventions, we discuss potential strategies to improve the effectiveness of tumor immunotherapy by re-educating TME-related factors.

## Current Status of Tumor Immunotherapy in Gastrointestinal Cancers

Immunotherapy has rapidly become a major treatment modality for several solid cancers, showing unprecedented efficacy. However, gastrointestinal tumors are considered as a “cold” malignancy due to the lack of effector T cell response and notoriously poor immunogenicity (Bonotto et al., 2017). Two PD1 blocking antibodies, pembrolizumab and nivolumab, have successfully achieved durable responses in some colorectal cancer patients (Procaccio et al., 2017). In addition, pembrolizumab has been approved by FDA for third-line treatment of PD-L1 positive advanced gastrointestinal tumors (Sun J. et al., 2020). Consistent with this, high expression of PD-L1 is associated with poor prognosis in gastrointestinal tumors (Sun J. et al., 2020). These studies suggest that immunotherapy approaches based on immune checkpoint inhibitor therapy have the potential to facilitate a paradigm shift in the treatment of gastrointestinal cancers and ultimately benefit patients with gastrointestinal tumors.

## Exosomes in Tumor Microenvironment of Gastrointestinal Cancers

Exosomes are extracellular vesicles with a membrane structure of 30–150 nm in diameter and are widely present in TME (Kowal et al., 2014). Exosomes secreted by tumor cells play a key role in signaling to neighbor cells, reshaping the TME, and promoting cell metastasis by carrying signaling peptides, non-coding RNAs and DNA, and immunostimulatory or inhibitory molecules (Simons and Raposo, 2009). In particular, exosomes are more stable in body fluids and their cargoes have the potential to be used as molecular markers for tumor diagnosis (Barile and Vassalli, 2017).

Recent studies have found that exosomes in TME are closely related to the effectiveness of tumor immunotherapy. Tumor-derived exosomes are capable of secreting and spreading immunosuppressive signals that modulate the proliferation and maturation of immune cells and ultimately their anti-cancer activity (Whiteside, 2017). PD-1 is the most important co-repressor signal and has been extensively studied for decades, with two ligands PD-L1 and PD-L2. Anti-PD-1 or PD-L1 has been a successful antitumor therapy strategy in recent years (Zappasodi et al., 2018). The aberrantly high expression of PD-L1 on the surface of tumor cells is the main mechanism by which tumor cells are able to evade immune surveillance. However, recent studies have revealed that cancer cells can secrete the vast majority of PD-L1 through exosomes, rather than presenting PD-L1 on the cell surface (Poggio et al., 2019). This is an interesting observation that well illustrates the phenomenon of systemic immune decline in tumor patients, i.e., tumor cells can cause distal immunosuppression through secreting PD-L1 containing exosomes. Interferon-gamma (IFN-γ) stimulation increases the amount of PD-L1 in tumor exosomes, which suppresses the function of CD8^+^ T cells and promotes tumor growth (Chen et al., 2018). Exosomal PD-L1 also has an immunosuppressive effect in gastric cancer and can predict patient prognosis (Fan et al., 2019). In addition to directly suppressing T cells in TME, exosomal PD-L1 can also be transferred to a variety of immune cells (Yang et al., 2018). In TME, exosomes secreted by PD-L1-expressing tumor cells can reach the draining lymph nodes and blood where they can inhibit T-cell activation (Chen et al., 2018). Not only tumor cells, but also multiple immune cells in TME express PD-L1 (Sun et al., 2018). Certain cytokines secreted by tumor cells promote the secretion of PD-L1 exosomes by non-tumor cells. For example, exosomes from bone marrow-derived cells (BMDCs) were found to carry PD-L1 in a tumor-bearing mouse model (Sun Y. et al., 2020). These observations suggest that exosomal PD-L1 is an important cause of immunosuppression in TME. The presence of exosomal PD-L1 makes it possible for tumors to suppress systemic immunity, and the suppressed immune environment further facilitates tumor cells proliferation and distal migration.

Tumor-secreted exosomes also play an important role in reshaping the microenvironment. Exosomes in the TME carry cargo to adjacent cells, especially immune cells, altering their biological properties to build an immunosuppressive microenvironment. In addition to T-cell immunity, innate immune cells are abundantly infiltrated in the TME, and in some cases, these immune cells determine the efficacy of tumor immunotherapy. For example, EBV-associated gastric cancer cells inhibit the maturation of dendritic cells by secreting exosomes, which leads to the invalidation of anti-tumor immunity (Hinata et al., 2020). In addition, extracellular vesicles of different cell sources carrying non-coding RNAs cause changes in downstream signaling pathways of target cells, which is another approach by the tumor cells to induce immune escape. In colorectal cancer, tumor cell-derived extracellular vesicles containing miR-424 inhibited the CD28-CD80/86 costimulatory pathway in tumor-infiltrating T cells and dendritic cells, a phenomenon that resulted an increase in the tumor’s response to immune checkpoint blockade therapies (Zhao et al., 2021). M1 macrophages are often considered to be a beneficial cell type for tumor immunotherapy, and recent studies have found that M1 macrophage-derived exosomes carrying miR-16-5p can promote T-cell immunity by reducing PD-L1 expression in gastric cancer cells (Li et al., 2020a). In contrast, miR-21-5p and miR-155-5p were highly expressed in the exosomes of M2 macrophages, mediating the migration and invasion of la colorectal cancer cells. miR-21-5p and miR-155-5p were transferred to colorectal cancer cells via exosomes and bound to the BRG1 coding sequence, downregulating the expression of BRG1, which is thought to be a key factor in promoting colorectal cancer metastasis (Lan et al., 2019). Colorectal cancer cell-derived exosomal miR-934 induces M2 macrophage polarization through downregulation of PTEN expression and activation of PI3K/AKT signaling (Zhao et al., 2020). In addition, a few miRNAs (miR-253p, miR-130b-3p, miR-425-5p) upregulated in colorectal cancer cells through activation of the CXCL12/CXCR4 axis, could be transferred to macrophages via exosomes. These exosomal miRNAs also regulate PTEN-induced macrophage M2 polarization through activation of PI3K/Akt signaling (Wang D. et al., 2020). Another non-coding RNA, lncRNA RPPH1, can be encapsulated in colorectal cancer cell-derived exosomes and mediates the polarization of macrophages toward M2 type (Liang et al., 2019). Neutrophils have been shown to promote immunosuppression in various cancers (Swierczak et al., 2015; Powell and Huttenlocher, 2016). Gastric cancer exosomes could prolong neutrophil survival and induce the expression of neutrophil inflammatory factors, which in turn promote migration of gastric cancer cells. Exosomes secreted by gastric cancer cells transport HMGB1 protein and induced neutrophil activation by interacting with TLR4 to activate the NF-κB pathway (Zhang et al., 2018). Gastric cancer-derived extracellular vesicles can induce neutrophil PD-L1 elevation through STAT3 pathway and inhibit T cell proliferation and function (Shi et al., 2020). Neutrophils are capable of secreting large amounts of inflammatory factors, and tumor-associated exosome regulation of neutrophils is particularly important in the remodeling of TME. In adaptive immunity, in addition to PD-L1, tumor cells secreted exosomes also can carry multiple non-coding RNAs to inhibit the function of cytotoxic T lymphocytes. A study found that colorectal cancer exosomes promote Th17 cell differentiation by delivering lncRNA CRNDE-h in TME (Sun et al., 2021). Th17 cells have both tumor-promoting and suppressive functions, which is a reflection of the complexity of the TME (Vitiello and Miller, 2020).

## Cancer-Associated Fibroblasts in Tumor Microenvironment of Gastrointestinal Cancers

Cancer-associated fibroblasts (CAF) are able to influence almost all malignant phenotypes of tumors by interacting with multiple cells in the TME through the constitutive secretion of cytokines, chemokines, metabolites, and stromal cell proteins (Pereira et al., 2019). Growing evidence suggests that CAFs contribute to tumor immune escape and immune checkpoint inhibitors resistance (Wong et al., 2019).

Cancer-associated fibroblasts elevate the expression of immune checkpoints in tumor cells. CAFs inhibit the proliferation and infiltration of CD8^+^ cytotoxic T lymphocytes in cancer through a high expression of presenilin 1 and promote tumor cell proliferation. Silencing presenilin 1 significantly reduced IL1β expression, and these effects were regulated through the WNT/β-catenin pathway (Zhang et al., 2020). The expression status of B7-H3 and α-SMA in CAFs could be used as a prognostic indicator for gastric cancer patients (Zhan et al., 2019).

Cancer-associated fibroblasts extracellular vesicles can transmit information between cells in the TME. A comprehensive proteomic analysis of CAFs-associated outer vesicles revealed that membrane-linked protein Annexin A6 effectively enhanced drug resistance in gastric cancer cells by activating β1 integrin-focal adhesion kinase (FAK)-YAP and inhibition of FAK or YAP may be a novel strategy for overcoming gastric cancer drug resistance (Uchihara et al., 2020). Meanwhile, YAP/TAZ upregulates PD-L1 expression in multiple cell types (Nguyen and Yi, 2019). YAP is a crucial regulatory molecule for CAFs, and activation of YAP is a hallmark feature of CAFs. In breast cancer, YAP is required for CAFs to promote stromal sclerosis, cancer cell invasion, and angiogenesis (Calvo et al., 2013). DKK3 links HSF1 and YAP/TAZ signaling to control the invasive behavior of CAFs in colorectal, ovarian and breast cancers (Ferrari et al., 2019). TGF-β stimulation of human lung-derived CAFs elevates PD-L1 expression through Smad2/3 and YAP/TAZ axis (Kang et al., 2020).

Cancer-associated fibroblasts are involved in the formation of the suppressive immune microenvironment. CAFs recruit immune cells by secreting large amounts of chemokines and inflammatory factors, such as chemokines CCL2, CXCL2, CXCL6, CXCL8, CXCL12, CXCL16, and inflammatory factors IL1b, S100A9, IL6 (Orimo et al., 2005; Tsuyada et al., 2012; Otomo et al., 2014; Allaoui et al., 2016; Yang et al., 2016; Kumar et al., 2017; Awaji et al., 2019). Aggregation of these immune cells in the tumor region was not able to hinder the growth of tumor cells, on the contrary, it was conditioned to become an inhibitory cell subpopulation. MMP9, CXCL1, CXCL2, CCL2, GM-CSF, IL-4, IL-6, and IL-8 produced by CAFs affect the polarization of macrophages toward M2 (Cohen et al., 2017; Cho et al., 2018; Kobayashi et al., 2019), and TGF-β secreted by CAFs is involved in neutrophil N2 polarization (Turley et al., 2015; Batlle and Massague, 2019). Both of these polarizations have a tumor-promoting function. Specifically, CCL2 and CCL8 can increase migration and invasion of colorectal cancer cells (Torres et al., 2013). See Table 1 for the complex mechanisms by which CAFs functions at TME. Moreover, CAFs produce multiple chemokines to induce the recruitment of polymorphonuclear MDSCs and Treg cells (Kumar et al., 2017). Furthermore, αSMA^+^ CAFs appeared to co-localize with FOXP3^+^ Treg in the TME, and CAFs recruited CD4^+^CD25^+^ T cells by secreting CCL5 and CXCL12 and increased their differentiation into tumor-promoting CD25^hi^FOXP3^hi^ Treg cells (Tan et al., 2011; Costa et al., 2018). CAFs expressing suppressive ligands could kill CD8^+^ T cells in an antigen-specific, antigen-dependent manner via PD-L2 and FASL. Thus, similar to the above, CAFs induce T cell suppression in the TME through a mechanism of action that relies on immune checkpoint activation and recruitment of immunosuppressive cells (Lakins et al., 2018).

**TABLE 1:** Molecules associated with CAFs in a multiplicity of tumors.

**Molecular**	**Tumor type**	**Note**	**References**
***Chemokines***			
CCL2	Breast cancer	Regulation of cancer stem cells	Tsuyada et al., 2012
CXCR2	Multiple cancers	Blocking granulocyte infiltration	Kumar et al., 2017
CXCL1	Multiple cancers	Recruiting granulocyte cells	Kumar et al., 2017
CXCL6	Lung cancer	Promoting tumor invasion	Otomo et al., 2014
CXCL8	Pancreatic tumor	Promoting tumor invasion	Awaji et al., 2019
CXCL12	Breast carcinoma	Promoting tumor growth	Orimo et al., 2005
CXCL16	Breast cancer	Attracting monocytes	Allaoui et al., 2016
GM-CSF	OSCC Colorectal cancer	Inducing of macrophage polarization	Cho et al., 2018
***Inflammatory factors***			
IL1b	Breast cancer	Promoting tumor progression	Lappano et al., 2020
IL6	Gastrointestinal cancer	Communication between mediated tumor cells and CAFs	Karakasheva et al., 2018
IL8	Gastric cancer	Mediating drug resistance	Zhai et al., 2019
TGF-β	Gastric cancer	Promoting tumor invasion	Ishimoto et al., 2017
S100A9	Colorectal cancer	Recruiting myeloid cells	Kim et al., 2012
MMP9	Breast cancer	Promoting tumor growth	Eiro et al., 2018
***Signaling pathways***			
WNT/β-catenin	Ovarian cancer	Regulation of tumor-infiltrating CTLs	Zhang et al., 2020
FAK-YAP	Gastric cancer	Enhancing Drug Resistance	Uchihara et al., 2020
YAP/TAZ	Colorectal cancer Breast cancer Ovarian cancer	Control the invasive behavior of CAFs	Ferrari et al., 2019

*CCL2, C-C Motif Chemokine Ligand; CXCL, C-X-C Motif Chemokine Ligand; GM-CSF, granulocyte macrophage colony-stimulating factor; OSCC, oral squamous cell carcinoma; MMP9, matrix Metallopeptidase 9. FAK, focal adhesion kinase; YAP, yes-associated protein; TAZ, transcriptional coactivator with PDZ-binding motif.*

That understanding of how CAFs modulate the TME will contribute to the development of tumor immunotherapeutic approaches and improve the effectiveness of immune checkpoint therapy and immune cell therapy. In addition to neutralizing important inhibitory cytokines in the TME, the search for cell-specific strategies to target CAFs is a promising means to re-educate the immune microenvironment.

## Nerves in Tumor Microenvironment of Gastrointestinal Cancers

Tumor-associated nerve fibers are considered to be components of the tumor mesenchyme and are involved in all stages of tumor development (Saloman et al., 2016). Neurotransmitters can modulate the immune response and thus influence the TME. Neurotrophic factors in the TME can directly stimulate cancer cells, induce tumor angiogenesis, and affect the prognosis of tumor patients (Magnon et al., 2013; Renz et al., 2018). With the growing evidence that neurogenesis promotes tumor progression, the role of neuronal cells in TME has to be taken into account, and targeting neuromodulator receptors may become a new avenue for anti-tumor therapy (Saloman et al., 2016).

The regulation of immunity by neuromodulator in the TME has been an important area in cancer biology (Dantzer, 2018). However, how the nervous system is involved in the tumorigenesis of gastrointestinal cancers has not been investigated sufficiently. The gastrointestinal tract is an organ system that receives a high degree of neural innervation and has a close connection with the central nervous system, as well as the existence of its own nervous system: the enteric nervous system (Furness, 2012). In the intestinal environment, progenitor cells of enteric nervous system origin have better migration, proliferation and neuronal differentiation capacity compared to cells of central nervous system origin (Findlay et al., 2014). The nervous system plays an important role in the regulation of epithelial homeostasis, and proper innervation is critical at all stages of gastric tumorigenesis (Zhao et al., 2014). Cancer stem cells from patients with gastric and colorectal cancers are capable of generating tumor neurons involved in tumor neurogenesis and proliferation. Knockout of the neurogenic capacity of human cancer stem cells could inhibit the growth of xenograft tumors (Lu et al., 2017). In colorectal cancer, the percentage of galanin-positive neurons observed in parts of the intestine without pathological changes was 35%, compared with 46% observed in the mesenteric plexus with pathological changes. Surgical or pharmacological removal of innervation significantly reduces tumorigenesis and progression (Godlewski and Pidsudko, 2012). This phenomenon is mediated through the vagus nerve via M3 receptors that regulate the Wnt signaling in tumor stem cells and are involved in gastric carcinogenesis (Zhao et al., 2014).

Neurogenic inflammation creates a microenvironment that is conducive to tumorigenesis. It is widely known that stress may lead to neurochemical changes, which can cause the release of several hormones that may promote cell proliferation and tumorigenesis. There are some examples where the use of neuromodulators, such as inhibitors of adrenergic signaling, appears to create an anti-tumor environment. Choline stimulates gastric epithelial cells to induce nerve growth factor (NGF) expression, and in turn, NGF overexpression in gastric epithelial cells amplifies intestinal nerves and promotes carcinogenesis. Ablation of Dclk1^+^ cells or blockade of the NGF/Trk signaling can inhibit epithelial cell proliferation and tumorigenesis in an acetylcholine muscarinic receptor 3 (M3R)-dependent manner, with the involvement of YAP (Hayakawa et al., 2017). Interestingly, upregulation of NGF mRNA resulted in an increased probability of developing rectal tumors in a colitis-associated cancer mouse model (Hayakawa et al., 2017). In pancreatic cancer, adrenergic signaling to tumor cells induces the release of NGF and brain-derived neurotrophic factor (BDNF), leading to an increase in nerve density in the tumor region. This in turn leads to an increase in adrenergic signaling, resulting in the accumulation of norepinephrine and enhanced tumor growth (Renz et al., 2018). NGF can also promote tumor cell proliferation, survival and metastasis in breast cancer (Adriaenssens et al., 2008). In addition to NGF, there are many types of neurotrophic factors in the microenvironment that can influence the interaction between epithelial and immune cells. Current evidence suggests that neuroactive drugs may influence the likelihood of cancer development through their neuromodulatory effects (Schonkeren et al., 2021).

A large variety of cytokines present in the TME can in turn affect the physiological activity of nerve fibers, such as macrophages released VEGF-A and IL1, which can regulate the activity and lengthening of adjacent nerve fibers and infiltration into the tumor (Lindholm et al., 1987; Cattin et al., 2015). M1 macrophages could destroy damaged nerves, whereas M2 macrophages are involved in nerve repair, possibly participating in tumor innervation. Semaphorin 4D could induce neurite growth and is mainly expressed in tumor-associated macrophages (TAM), which may be involved in tumor innervation (Capparuccia and Tamagnone, 2009). Glucocorticoid-induced β-adrenergic receptors (β2-AR) expression could lead to a decrease in the number of NK and NKT cells and a decrease in cytotoxic activity (De Lorenzo et al., 2015). In adaptive immunity, activation of the β-AR signaling pathway significantly inhibited CD8^+^ T cell production, proliferation (Nissen et al., 2018). β2-AR interacted with chemokine receptors CCR7 and CXCR4 to promote lymphocyte retention in lymph nodes; activation of β2ARs enhanced retention-promoting signaling through CCR7 and CXCR4, inhibiting lymphocyte export in lymph nodes (Nakai et al., 2014; Wang W. et al., 2020). Those observations suggested interactions between the nerves and the immune cells, although direct evidence for this interaction has not been obtained. Nerves are important members of the TM E in gastrointestinal tumors, however, the existed studies are still limited, especially, the role of neurons in the tumorigenesis are still poorly understood.

## Cytokines in Tumor Microenvironment of Gastrointestinal Cancers

A multitude of inflammatory cytokines are present in TME of gastrointestinal cancers, and orchestrate the process of anti-tumor immunity, especially in inflammation-associated colorectal cancers (Chen et al., 2017). At the same time, cancer cells also hijack inflammatory pathways to suppress tumor immunity by enhancing PD-L1 expression and reshaping the immune microenvironment to create favorable conditions for tumor progression. Here, we highlight several cytokines that are closely related to tumor immunotherapy, including IFN-γ, IL6, IL17, TGF-β. Almost every cytokine is more or less involved in immune regulation in the microenvironment, and the cytokines in TME may be considered as a whole for tumor immunotherapy in the future.

## IFN-γ

IFN-γ was initially considered as a classical pro-inflammatory cytokine involved in the regulation of anti-inflammatory responses by antagonizing IL-10 (Herrero et al., 2003) and TGF-β (Park et al., 2007) signaling. IFN-γ is essential for inducing the proliferation of cytotoxic T cell precursors, and also, IFN-γ is a “weapon” for cytotoxic CD8^+^ T cells, whose activation status is represented by the expression of IFN-γ in CD8^+^ T cells (Curtsinger et al., 2012; Chen et al., 2018). IFN-γ also mediates the anti-tumor immune response of Th1 cells (Haabeth et al., 2011). IFN-γ also can induce the classical activation pathway of macrophages, polarizing them toward M1-type, exhibiting enhanced phagocytosis and increased secretion of cytokines (Nathan et al., 1983; Sadlik et al., 1985). However, under some chronic inflammatory conditions, IFN-γ may cause immunosuppression. In such cases, IFN-γ may play a protective role by increasing the number and function of Treg cells (Nishibori et al., 2004). Interestingly, IFN-γ is a broad-spectrum PD-L1 inducer, capable of elevating PD-L1 expression in a variety of tumor and immune cells (Sun et al., 2018) and measuring IFN-γ expression levels in the microenvironment allows for a rough determination of PD-L1 abundance (Chen et al., 2018).

## IL-6

IL-6 is the most important acute inflammatory factor and also plays an important role in TME. IL-6 is a pleiotropic cytokine that is widely recognized as a major regulator of the acute phase response and regulates the immune response by activating the JAK/STAT pathway (Hunter and Jones, 2015). In TME, IL-6 is a major contributor to the dynamic cross-talk between tumor cells and CAFs (Karakasheva et al., 2018). Recent studies have demonstrated that the pro-inflammatory cytokine IL-6, produced in the tumor-bearing state, is associated with promotion of metastatic colonization of colon cancer cells with dysfunctional anti-tumor immunity. In IL-6-deficient mice, CT26 cells showed reduced metastatic colonization in the liver, enhanced antitumor effector function of CD8^+^ T cells, and enhanced IL-12 production by CD11c^+^ dendritic cells. *In vivo* injection of anti-PD-L1 effectively inhibited the metastatic colonization of CT26 cells in IL-6-deficient mice (Toyoshima et al., 2019). Furthermore, IL-6-activated JAK1 phosphorylates PD-L1 Tyr112, which activates the endoplasmic reticulum-associated *n*-glycosyltransferase STT3A to catalyze PD-L1 glycosylation and maintain PD-L1 stability (Chan et al., 2019). This study reveals the mechanism by which IL-6 regulates the initiation of PD-L1 glycosylation, providing more possible ways to target PD-L1. Inhibition of IL-6Ra and downstream signaling pathways provides the basis for a novel targeted therapy for oral upper gastrointestinal cancers.

## IL-17

IL-17 is mainly produced by activated Th17 cells and is associated with Treg cell differentiation. IL-17 signaling to tumor cells upregulates PD-L1 levels in these cells, thereby supporting their resistance to immune destruction (Wang et al., 2017). IL-17 also recruits MDSCs to accumulate in the TME, thereby inhibiting anti-tumor immune activity (Wang et al., 2014). Not only a direct mechanism of tumor promotion, but IL-17 also leads to colorectal cancer resistance to anti-angiogenic therapy (Chung et al., 2013). IL-17 upregulates PD-L1 protein in HCT116 cells through activation of NF-κB and ERK1/2 signaling (Wang et al., 2017). However, in patients with resected gastric cancer, the number of Th17 cells decreased whereas the number of Treg cells and PD1/PD-L1 expression increased (Zheng et al., 2019). Consistent with this, in patients with hepatocellular carcinoma, inflammatory cytokines released from IL-17-activated monocytes stimulate PD-L1 expression and effectively suppress cytotoxic t-cell immunity *in vitro* (Zhao et al., 2011). The pro-inflammatory properties of IL-17 are critical for its host protective capacity, but unrestrained IL-17 signaling is associated with immunopathology, autoimmune disease, and cancer progression (Amatya et al., 2017). IL-17 tends to perform different functions in different microenvironments, making the immune status of TME more complex. IL-23 is thought to be the basic cytokine driving IL-17 production by T cells, and IL-1 synergizes with IL-23 to promote IL-17A production by T cells (Mills et al., 2013). IL-23 can also be produced by dendritic cells and is involved in the polarization response of T cells (Roses et al., 2008). IL-23 supports skin cancer development by inducing IL-17A and MMP9 expression, reducing CD8^+^ T cell infiltration into tumors by activating the STAT3 pathway in tumors and stromal cells, and also promoting myeloid cell infiltration into the TME (Langowski et al., 2006).

## TGF-β

TGF-β is a major inducer of epithelial-mesenchymal transition (EMT) in epithelial tumor cells (Massague, 2008). TGF-β is also a major immunomodulatory factor and its immunosuppressive effects in the microenvironment are analyzed here. TGF-β is usually considered as an anti-inflammatory cytokine and the association with PD-L1 seems to make sense in terms of its function (Tauriello et al., 2018). Almost all nucleated cells produce TGF-β and respond to it, and among the mechanisms used by cancer cells to evade immune surveillance, TGF-β production is thought to be the most effective (Larson et al., 2020). Blockade of TGF-β downregulated PD-1 and PD-L1 expression, which is consistent with the phenotype of PD-L1 expression by dendritic cells stimulated *in vitro* using TGF-β (Song et al., 2014). TGF-β effectively increased the expansion of Treg cells and upregulated dendritic cells PD-L1 expression. TGF-β also can induce PD-L1 expression in fibroblasts via Smad2/3 and YAP/TAZ axis (Kang et al., 2020). Increased TGF-β in TME represents a major immune evasion mechanism that promotes T-cell depletion and renders tumors insensitive to anti-PD-1-PD-L1 antibody therapy (Tauriello et al., 2018). Therapeutic co-administration of TGF-β-blocking and anti-PD-L1 antibodies reduced TGF-β in stromal cells, facilitated T-cell penetration, and induced vigorous anti-tumor immunity (Mariathasan et al., 2018).

It is important to recognize that the effect of inflammatory factors on PD-L1 expression may be context-dependent. There is evidence that PD-L1 expression on tumor cells and immune cells can be differentially regulated. For example, in a mouse cancer model, treatment of mice with IFN-γ-blocking antibodies largely abolished PD-L1 expression on tumor cells but only partially reduced PD-L1 levels on tumor-associated macrophages (Noguchi et al., 2017). Our study found that the inflammatory factor S100A8 promoted PD-L1 expression on macrophages, but not on tumor cells (Li et al., 2020b). This illustrates the cell-specific nature of inflammatory factors affecting PD-L1 expression, which may be related to the expression of different types of cell surface receptors or may be resulted from the wide variation in epigenetic modifications of histones in different cell types.

## Metabolites in Tumor Microenvironment of Gastrointestinal Cancers

Metabolic reprogramming of tumor cells is one of the top ten features of tumor cells (Hanahan and Weinberg, 2011), and several metabolic enzymes are valuable drug targets for the treatment of cancer (Luengo et al., 2017). In TME, in addition to the intrinsic metabolism of tumor cells, competition and crosstalk between different cell types lead to a complex metabolic environment (Elia and Haigis, 2021). Nutrient depletion and hypoxia occur due to the high utilization of nutrients and oxygen by tumor cells, and this variation may depend on tumor type and tumor location (Sullivan et al., 2019). Enhanced aerobic glycolysis, also known as the Warburg effect, has emerged as the clearest and most widespread metabolic adaptation to maintain cancer.

Oxygen is a key factor in the energy source required for mitochondrial function and differentiation of immune cells, and alleviating hypoxia may promote antitumor immunity and enhance the response to anticancer immunotherapy. It has been shown that supplemental oxygen reduces immunosuppressive adenosine levels and increases CD8^+^ T cell infiltration and inflammatory cell levels in lung tumor-bearing mice (Hatfield et al., 2015). In addition to oxygen, highly proliferative activated T cells are heavily dependent on glucose metabolism. When the availability of glucose in TME is reduced due to high uptake by cancer cells, CD8^+^ T cells exhibit very low infiltration and proliferation (Singer et al., 2011).

In such a complex metabolic microenvironment, studies have identified many metabolites that interfere with the antitumor immune response. For example, in colorectal tumor-bearing mice, blocking glutamine inhibited oxidative and glycolytic metabolism of cancer cells, leading to hypoxia, acidosis, and reduced nutrient consumption. Effector T cells respond to glutamine antagonism by significantly upregulating oxidative metabolism, with increased T cell lifespan and a highly activated phenotype (Leone et al., 2019). Methionine is an essential amino acid that plays an important role in T-cell differentiation. Recent studies have found that human colorectal tumor cells have higher expression of the methionine transporter SLC43A2 than T cells, and that cells take up large amounts of methionine from TME, which blocks T cell differentiation (DePeaux and Delgoffe, 2021). Methionine also maintains the epigenetic adaptations required for Th17 cell proliferation and cytokine production (Roy et al., 2020). Arginase 1 (ARG1) is expressed by myeloid-derived cells in TME, particularly M2 macrophages, and can reduce arginine levels in TME. Arginine deficiency reduces T-cell proliferation and enhances tumor immune effects when ARG1 inhibitors are used in combination with PD1 inhibitors (Grzywa et al., 2020). Lactate production by tumor cells has an important role in signaling and TAM polarization. Tumor cell-produced lactate can promote the macrophage M2 phenotype by stabilizing hypoxia-inducible factor 1-alpha (HIF-1α) and activating g protein-coupled receptor 132 (GPR132) to induce vascular endothelial growth factor (Colegio et al., 2014). Additional studies have found that epigenetic modifications in M1 macrophages are also associated with lactate metabolism (Zhang et al., 2019).

Immune cells require adequate nutrition to function properly, which is obviously difficult to maintain in TME. Thus, a better understanding of the different metabolites between tumor cells and immune or stromal cells could provide a unique therapeutic window for metabolic therapies. Considering that most metabolites are not toxic to normal tissues, metabolic therapies may be powerful adjuvant to tumor immunotherapy.

## Microbiome of Gastrointestinal Cancers

The human organism is home to trillions of microorganisms, as many as human cells, which constantly interact with the host at many sites (including the skin and mucosal surfaces, such as the gastrointestinal tract and lung) (Sender et al., 2016; Gopalakrishnan et al., 2018a). There is a growing body of evidence supporting the role of the microbiome in response to cancer therapy, particularly to immune checkpoint inhibitors that span multiple cancer types. The overall gut microbiome composition of cancer patients differs from that of healthy individuals, which may contribute to the effectiveness of immune checkpoint therapy (Zackular et al., 2014; Chaput et al., 2017). The immune microenvironment of gastrointestinal tumors is directly affected by the gut microbes’ metabolites. One mechanism is that dendritic cells, when carrying antigens from bacteria in the draining lymph nodes of the intestine, can stimulate B and T cells circulating with the blood, including Treg and Th17 cells, activating systemic immunity or promoting immune responses to other antigens through cross-reactivity with similar antigenic epitopes (Stary et al., 2015).

Researchers found significant differences in tumor growth rates and a number of infiltrating immune cells in mouse melanomas from the Jackson lab and the Taconic Farms lab. This is an interesting phenomenon, for which we would normally choose to explain this phenomenon in terms of genetic differences, however, these differences were eliminated after co-habitation. Fecal transplantation experiments in mice delivered the answer: the differences in immunotherapy in different lab mice come from differences in the tumor and number of gut microbes. The authors identified *Bifidobacterium bifidum* as having anti-tumor effects, and combination with PD-L1-blocking antibodies improved the therapeutic effect (Sivan et al., 2015). Researchers also found that the immune system was defective, the mucus layer was lost and the size and function of the draining mesenteric lymph nodes were reduced in germ-free mice (Spiljar et al., 2017). A recent study isolated 11 bacterial strains from healthy human donor feces that is capable of inducing large numbers of IFN-γ^+^ CD8^+^ T cells in the intestine. When used in combination with immune checkpoint inhibitors, these bacterial strains were able to inhibit tumor growth (Tahara et al., 2014; Saito et al., 2016; Tanoue et al., 2019).

The studies presented above have found that the response to immune checkpoint inhibitors is influenced by the composition of the gut microbiota. The overall and progression-free survival rates were significantly higher in cancer patients not treated with antibiotics for conventional indications under anti-PD-1/PD-L1 therapy than in patients treated with antibiotics. This interesting phenomenon suggested that the use of antibiotics may disrupt the gut microbiota and thus impair the response to anti-tumor immunity and immune checkpoint blockade (Routy et al., 2018). *Bifidobacteria* combined with anti-PD-L1 treatment almost completely alleviated tumor growth, an effect that was mediated by increased dendritic cell function and mediated the anti-tumor effect of CD8^+^ T cells (Sivan et al., 2015; Vetizou et al., 2015). Patients who received anti-PD-1 treatment showed a significant increase in intestinal flora diversity with relative abundance of certain microorganisms, such as *Clostridiales*, *Ruminococcaceae*, and *Faecalibacterium* (Gopalakrishnan et al., 2018b).

Intestinal flora are xenobiotics for the body and are recognized as pathogen-associated molecular pattern (PAMPs) by pattern recognition receptors (PRRs) like Toll-like receptors (TLRs) or other receptors on the surface of antigen-presenting cells (e.g., macrophages, dendritic cells, B cells). Activation of TLR signaling in antigen-presenting cells causes activation of MyD88 and downstream TRIF6 molecules, nuclear entry of NF-κB, AP-1 and IRF3 transcription factors, and massive expression of cytokines such as TNF-α, IL-6, and IFN-γ (Kawai and Akira, 2007). activation of TLR-3/4 also leads to increased expression of PD-L1 and suppression of antitumor immunity (Lu et al., 2008; Pulko et al., 2009; Li et al., 2020b). This may be one of the mechanisms by which gut microbes can influence the effect of tumor immunotherapy at other distal sites.

## Conclusion

The efficacy of immunotherapy for gastrointestinal tumors is influenced not only by genomic aberrations in the tumor cells themselves, but also by the modulation of TME. Numerous studies have identified TME remodeling as one of the important causes of tumor immunotherapy tolerance. Clinical observations and pathological studies have identified non-immune cell components in the TME that also have a profound impact on tumor immunotherapy, and these non-immune cell components should not be neglected. Here, we reviewed the mechanisms of several important non-immune cell components in TME involved in immune regulation and their complex regulatory relationships with TME (Figure 1).

Although significant progress has been made in this area, there are still some issues that need to be addressed in the near future. non-immune components of TME are complexly regulated and can generate complex regulatory networks with immune cells, and it is easy to attain one thing and lose sight of another. Although single-cell sequencing has greatly improved the accuracy of the analysis of the cellular components of TME, it is difficult to distinguish the non-cell components. In addition, TME non-immune cells are also heterogeneous and plastic, for example, the heterogeneity of CAFs has led to the failure of early clinical trials targeting CAFs. Overcoming TME heterogeneity to find representative targeting molecules is the key to address tumor immunotherapy tolerance. Exosomes and cytokines are natural tumor markers that have access to the circulatory system, and their expression can influence the efficacy of tumor immune checkpoint therapy, which has important implications for predicting the prognostic outcome of treatment. The microbiome has been well documented to influence tumor immunotherapy through its metabolites. Identification of specific flora of microbiome that promote immunotherapy and re-establishment of flora types in cancer patients is expected to prevent tumor recurrence. However, there are confounding factors that affect the patient’s microbiome, including different diets, antibiotic medications, emotional factors, and the influence of environmental microorganisms. These factors make it difficult to re-establishing “health” microbes in patients. Discovery or modification of powerful, interference-resistant strains to enhance tumor immunotherapy holds great promise. Non-immune cell components play an important role in immunotherapy of gastrointestinal tract tumors, both promotive and antagonistic. It is imperative to elucidate the role of more molecules and cell types in TME to provide sufficient theoretical guidance for targeting TME and to better facilitate drug design and development. In conclusion, for tumor immunotherapy, reprogramming immune cells to change their type and function to fight against tumor cells using the plasticity of TME may be one of the most effective ways to enhance the effectiveness of tumor immunotherapy in the coming years.

## Author Contributions

ZL and JM: methodology and writing. XZ and CL: methodology. All authors contributed to the article and approved the submitted version.

## Conflict of Interest

The authors declare that the research was conducted in the absence of any commercial or financial relationships that could be construed as a potential conflict of interest.

## Publisher’s Note

All claims expressed in this article are solely those of the authors and do not necessarily represent those of their affiliated organizations, or those of the publisher, the editors and the reviewers. Any product that may be evaluated in this article, or claim that may be made by its manufacturer, is not guaranteed or endorsed by the publisher.

## Abbreviations

TME: tumor microenvironment
Treg: regulatory T
MDSC: myeloid-derived suppressor cells
MSI: microsatellite instability
dMMR: mismatch repair-deficient
CAF: cancer-associated fibroblasts
NGF: nerve growth factor
TLRs: toll-like receptors.
